# Supplementary material for: Auditory-motor synchronization and perception suggest partially distinct time scales in speech and music
Source: Commun Psychol. 2024 Jan 3;2:2. doi: 10.1038/s44271-023-00053-6 (PMC11332030; doi:10.1038/s44271-023-00053-6)
Supplement: Supplementary file 1 — Supplemental Material [file 44271_2023_53_MOESM1_ESM.pdf]

Supplementary Material

Auditory-Motor Synchronization and Perception Suggest Partially Distinct Time Scales in Speech and Music

Alice Vivien Barchet[1*], Molly J. Henry[2,3], Claire Pelofi[4,5] and Johanna M. Rimmele[1,5*]

Affiliations:

[1] Departments of Neuroscience and Cognitive Neuropsychology, Max Planck Institute for Empirical Aesthetics, 60322 Frankfurt am Main, Germany

[2] Research Group 'Neural and Environmental Rhythms', Max Planck Institute for Empirical Aesthetics, 60322 Frankfurt am Main, Germany

[3] Department of Psychology, Toronto Metropolitan University, Toronto, Canada

[4] Music and Audio Research Laboratory, New York University, New York, NY, USA

[5] Max Planck NYU Center for Language, Music, and Emotion, Frankfurt am Main, Germany, New York, NY, USA

Corresponding authors: Alice Vivien Barchet (alice-vivien.barchet@ae.mpg.de) and Johanna M. Rimmele (johanna-rimmele@ae.mpg.de)

Supplementary Material

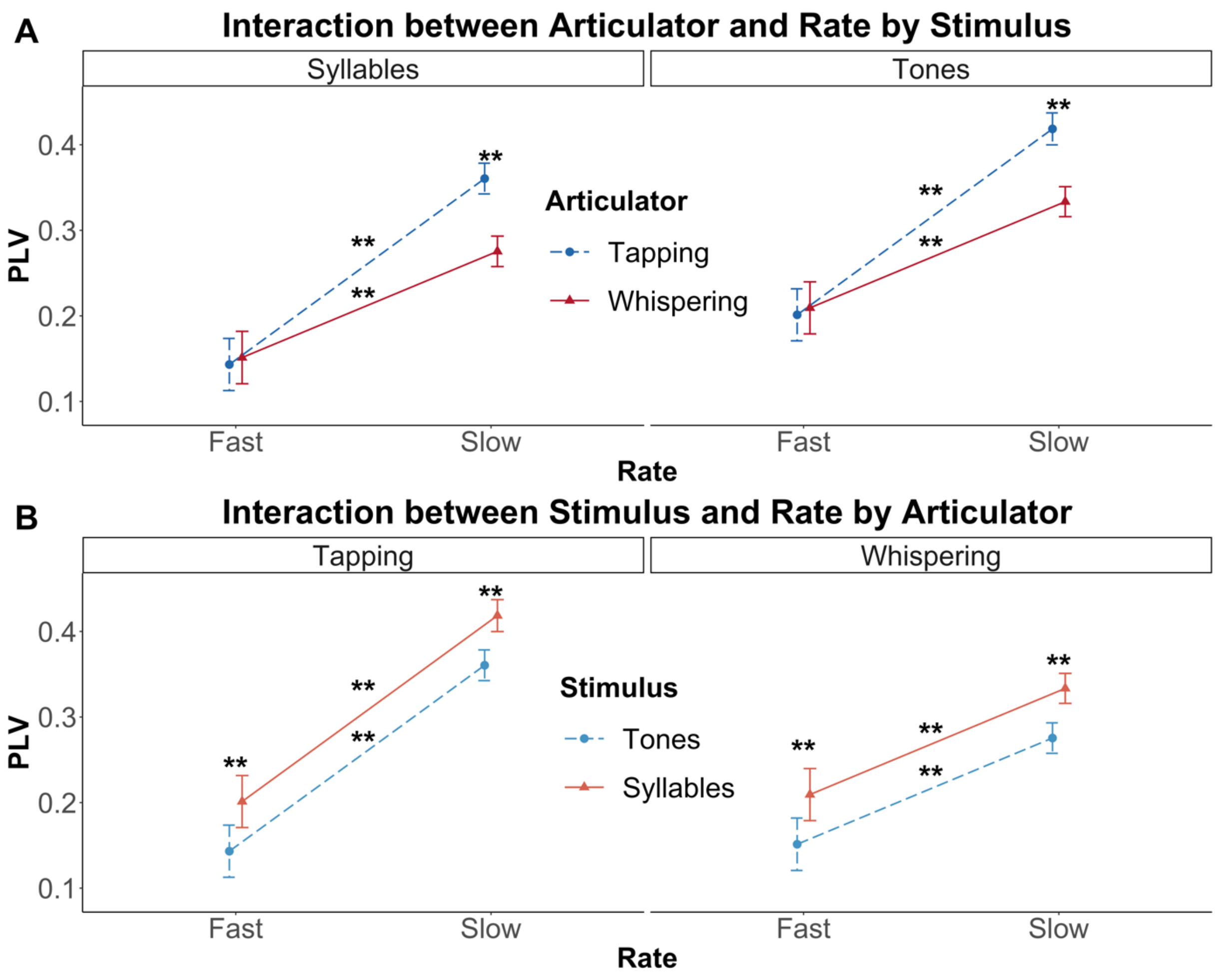

Figure S1. Model Output for the Linear Mixed Effects Model for the Synchronization Task.

Error bars show the 95% confidence interval.

** $p < .01$

Supplementary Note 1

Order Control Analysis

In the synchronization task, participants complete a total of 8 different conditions, leading to 16 subsequent synchronization trials. Due to the considerable length of the synchronization part, we considered that participants might, on the one hand, experience fatigue effects over the course of the experiment. On the other hand, participants might be influenced by practice effects across the 8 conditions, leading to an improved performance over the course of the experiment.

To exclude effects of the condition order in the synchronization task, we included the condition order in the mixed effects model for the synchronization data. As displayed in supplementary table 1, the analysis revealed that the condition order did not have a significant effect on synchronization performance.

Supplementary Table 1

Results of the Linear Mixed Effects Model for the Synchronization Task while Controlling for Condition Order

Effect	Estimate	95%CI	Partial η^2	df	p
Intercept	0.13	[0.10, 0.17]	--	116.53	< .001 **
Tempo (slow)	0.22	[0.19, 0.25]	0.73	112.25	< .001 **
Motor effector (whisper)	0.01	[-0.01, 0.03]	0.07	367.87	.584
Stimulus (tones)	0.06	[0.04, 0.07]	0.14	370.63	< .001 **
Tempo x Effector	-0.09	[-0.12, -0.06]	0.10	371.41	< .001 **
Motor envelope width	0.03	[0.02, 0.04]	0.05	443.23	. < .001 **
Condition Order	0.003	[0.00, 0.01]	0.01	399.69	.096

** p < .01

The Influence of Musical Sophistication

Auditory perception and synchronization performance are likely to be driven by musicality. We therefore controlled the synchronization and the perception model for self-reported musical experience as assessed by the Gold-MSI. Results for the synchronization model are displayed in supplementary table 2. Musical sophistication had a significant positive effect on synchronization performance and it interacted with the rate and the modality. Interestingly, effects of musical experience on synchronization performance were stronger in fast rates than in slow rates. Additionally, the effect of musical experience was stronger in whispering than in tapping. No interaction between musical experience and the stimulus type was detected.

Supplementary Table 2

Results of the Linear Mixed Effects Model for the Synchronization Task while Controlling for Characteristics of the Envelope

Effect	Estimate	95%CI	Partial η^2	df	p
Intercept	0.14	[0.12, 0.17]	--	92.88	< .001 **
Rate (slow)	0.22	[0.19, 0.25]	0.75	115.61	< .001 **
Effector (whisper)	0.01	[-0.01, 0.03]	0.06	367.24	.428
Stimulus (tones)	0.06	[0.04, 0.07]	0.14	370.45	< .001 **
Rate x Effector	-0.09	[-0.12, -0.06]	0.10	371.18	< .001 **
Motor Envelope Width	0.03	[0.02, 0.04]	0.05	443.21	< .001 **
MSI (global)	0.04	[0.02, 0.07]	0.26	70.33	.002 **
Rate x MSI (global)	-0.03	[-0.06, -0.01]	0.10	61.18	.012 *
Effector x MSI (global)	0.02	[0.00, 0.03]	0.02	366.57	.017 *

** p < .01

* p < .05

When correlating the 3 PCA components with the global score of the Gold-MSI, we revealed that self-reported musicality was highly correlated with the fast PCA component, r = .44, p < .001, 95% CI [.21, .62]. Musicality additionally correlated with the slow whispering component, r = .37, p = .003, 95% CI [.13, .57]. Musicality was uncorrelated with the slow tapping component, r = -.09, p = 0.48, 95% CI [-.33, .16].

To investigate if synchronization performance influences perception performance independent of musicality, we included the standardized global score of the Gold-MSI in a control model of the logistic mixed effects model predicting the perception performance.

The results of the control model including the MSI global score are displayed in supplementary table 4. The global MSI score did not significantly predict perception performance. Thus, the synchronization components predicted perception performance independent of self-reported musical sophistication.

Supplementary Table 3

Results of the Generalized Logistic Mixed Effects Model for the Perception Task while Controlling for Self-Reported Musical Sophistication

Effect	Estimate	95%CI	Odds Ratio	p
Intercept	1.92	[1.70, 2.14]	6.80	< .001 **
Stimulus (syllables)	-0.65	[-0.90, -0.40]	0.52	< .001 **
Rate (slow)	-0.24	[-0.51, 0.03]	0.79	.087
Rate x Stimulus	1.29	[0.86, 1.73]	3.64	< .001 **
Fast component	0.34	[0.17, 0.52]	1.41	< .001 **
Slow tapping component	0.22	[0.07, 0.37]	1.25	.005 **
Motor envelope width	-0.20	[-0.28, -0.13]	0.82	< .001 **
MSI (global)	0.02	[-0.15, 0.19]	1.02	.782

** p < .01

Supplementary Note 2

Deviations from the Preregistration

We adjusted the preregistered protocol in several necessary aspects. Concerning the planned analyses, we decided to analyze the synchronization data using a linear mixed model instead of a repeated measures ANOVA, as stated in the preregistration. We decided to use a linear mixed model since this method allowed us to control the model for the auditory and the motor envelope characteristics. Additionally, we could directly compare the main model with the model controlled for the condition order. Controlling for these continuous variables would not have been possible in an ANOVA architecture. We report the results of the ANOVA below. Due to the lack of control for envelope characteristics, an interaction between stimulus type and rate as well as a three-way interaction between rate, motor effector, and stimulus type reached significance.

Supplementary Table 4

Results of the preregistered ANOVA for the synchronization task.

Effect	F	Dfn	Dfd	p	Partial η^2	95%CI
Intercept	779.33	1	61	< .001 **	0.93	[0.89, 0.95]
Rate	183.07	1	61	< .001 **	0.75	[0.63, 0.81]
Motor effector	25.07	1	61	< .001 **	0.29	[0.11, 0.45]
Stimulus	97.21	1	61	< .001 **	0.61	[0.45, 0.71]
Rate x Effector	41.76	1	61	< .001 **	0.41	[0.22, 0.55]
Rate x Stimulus	4.2	1	61	0.045 *	0.06	[0.00, 0.21]
Effector x Stimulus	1.28	1	61	0.263	0.02	[0.00, 0.13]
Rate x Effector x Stimulus	4.96	1	61	0.03 *	0.08	[0.00, 0.22]

** p < .01

* p < .05

Additionally, we preregistered that we planned to recruit a sample of N = 55 participants. However, since early results indicated that several exclusions might be necessary, we decided to collect the data of eleven additional participants so that the inclusion of approximately 55 participants in all analyses was possible. Concerning the exclusion criteria, we considered the updated exclusion criteria for the SSS-Test (Lizcano-Cortés et al., 2022) that were not available at the time of the preregistration. This led to the additional exclusion criterium of consistency between the two synchronization trials, that we adhered to. Due to the number of participants already excluded, we decided not to exclude additional participants based on

outlier behavioral performance in the perception task as preregistered. However, the analyses for the perception task were repeated excluding the 3 participants falling below the median – 2.5 absolute deviations limit defined in the preregistration with no major difference in the results. The results for the generalized linear mixed effects models for the perception task excluding the three participants with outlier behavioral performance are displayed in supplementary table 5.

Supplementary Table 5

Results for the Perception Task with the preregistered exclusion criteria.

Effect	Estimate	95%CI	Odds Ratio	p
Intercept	2.02	[1.8, 2.24]	7.53	< .001 **
Rate (slow)	-0.24	[-0.53, 0.05]	0.79	0.102
Stimulus (tones)	-0.69	[-0.96, -0.42]	0.5	< .001 **
Rate x Stimulus	1.37	[0.91, 1.84]	3.95	< .001 **
Fast component	0.32	[0.18, 0.46]	1.38	< .001 **
Slow tapping component	0.17	[0.03, 0.31]	1.19	0.016 *
Auditory envelope width	-0.23	[-0.31, -0.16]	0.79	< .001 **

Three additional participants were excluded due to outlier behavioral performance as defined in the preregistration. N = 54

** p < .01

* p < .05
