# Supplementary material for: Auditory-motor synchronization and perception suggest partially distinct time scales in speech and music
Source: Commun Psychol. 2024 Jan 3;2:2. doi: 10.1038/s44271-023-00053-6 (PMC11332030; doi:10.1038/s44271-023-00053-6)
Supplement: Supplementary file 3 — Reporting Summary [file 44271_2023_53_MOESM3_ESM.pdf]

nature portfolio

Corresponding author(s): Alice Vivien Barchet & Johanna M. Rimmele

Last updated by author(s): Dec 11, 2023

Reporting Summary

Nature Portfolio wishes to improve the reproducibility of the work that we publish. This form provides structure for consistency and transparency in reporting. For further information on Nature Portfolio policies, see our Editorial Policies and the Editorial Policy Checklist.

Statistics

For all statistical analyses, confirm that the following items are present in the figure legend, table legend, main text, or Methods section.

n/a	Confirmed	
☐	☒	The exact sample size (*n*) for each experimental group/condition, given as a discrete number and unit of measurement
☐	☒	A statement on whether measurements were taken from distinct samples or whether the same sample was measured repeatedly
☐	☒	The statistical test(s) used AND whether they are one- or two-sided<br>*Only common tests should be described solely by name; describe more complex techniques in the Methods section.*
☐	☒	A description of all covariates tested
☐	☒	A description of any assumptions or corrections, such as tests of normality and adjustment for multiple comparisons
☐	☒	A full description of the statistical parameters including central tendency (e.g. means) or other basic estimates (e.g. regression coefficient) AND variation (e.g. standard deviation) or associated estimates of uncertainty (e.g. confidence intervals)
☐	☒	For null hypothesis testing, the test statistic (e.g. *F*, *t*, *r*) with confidence intervals, effect sizes, degrees of freedom and *P* value noted<br>*Give P values as exact values whenever suitable.*
☐	☒	For Bayesian analysis, information on the choice of priors and Markov chain Monte Carlo settings
☐	☒	For hierarchical and complex designs, identification of the appropriate level for tests and full reporting of outcomes
☐	☒	Estimates of effect sizes (e.g. Cohen's *d*, Pearson's *r*), indicating how they were calculated

Our web collection on statistics for biologists contains articles on many of the points above.

Software and code

Policy information about availability of computer code

Data collection	We used custom code relying on the Psychophysics Toolbox Version 3.0.12 running on MATLAB version R2021a. Questionnaire data was collected using LimeSurvey.
Data analysis	We used custom code relying on the Neural Systems Laboratory (NSL) Auditory Model toolbox running on MATLAB version 9.9.0.1592791 (R2020b) for the analysis of the auditory envelopes. For the PLV calculation, we used scripts provided by Lizcano-Cortés et al. available at https://doi.org/10.5281/zenodo.6142988.<br><br>Additionally, R version 4.0.5 running on RStudio version 1.4.1106 was used for statistical analysis, relying on the packages lme4 version 1.1-28, lmerTest version 3.1-3, psych version 2.3.9, car version 3.1-0, emmeans version 1.7.2, DHARMa version 0.4.6, effectsize version 0.8.6, performance version 0.10.5, MVN version 5.9, BFDA version 0.5.0. The plots were created using ggplot2 version 3.4.4 , sjPlot version 2.8.15 as well as introdataviz version 0.0.0.9003.

For manuscripts utilizing custom algorithms or software that are central to the research but not yet described in published literature, software must be made available to editors and reviewers. We strongly encourage code deposition in a community repository (e.g. GitHub). See the Nature Portfolio guidelines for submitting code & software for further information.

Data

Policy information about availability of data

All manuscripts must include a data availability statement. This statement should provide the following information, where applicable:

- Accession codes, unique identifiers, or web links for publicly available datasets
- A description of any restrictions on data availability
- For clinical datasets or third party data, please ensure that the statement adheres to our policy

The anonymized data including responses in the perception task as well as questionnaire responses have been deposited at https://osf.io/9qthr/. Additionally, the repository contains the baseline corrected PLVs. Raw audio recordings cannot be provided for data protection reasons, instead we provide them as processed data (i.e., envelopes).

Human research participants

Policy information about studies involving human research participants and Sex and Gender in Research.

Reporting on sex and gender	Gender information was assessed using self-report. We asked participants for their “Geschlecht”. In German, there is no clear distinction between sex and gender, but we assume that the participants reported their societal gender. Participants did not have to reveal their gender indentification if they did not feel comfortable doing so. The complete sample included 38 women, 23 men, 2 non-binary, and 1 undisclosed gender. Gender-based effects were not deemed relevant for the study's aim and thus we performed no gender-based analyses.
Population characteristics	See below.
Recruitment	Participants were recruited from the participant database of the Max Planck Institute for Empirical Aesthetics.
Ethics oversight	All experimental procedures were ethically approved by the Ethics Council of the Max Planck Society (Nr. 2017_12).

Note that full information on the approval of the study protocol must also be provided in the manuscript.

Field-specific reporting

Please select the one below that is the best fit for your research. If you are not sure, read the appropriate sections before making your selection.

☐ Life sciences ☒ Behavioural & social sciences ☐ Ecological, evolutionary & environmental sciences

For a reference copy of the document with all sections, see nature.com/documents/nr-reporting-summary-flat.pdf

Behavioural & social sciences study design

All studies must disclose on these points even when the disclosure is negative.

Study description	The study includes quantitative behavioral data.
Research sample	The sample included German native speakers recruited in the area of Frankfurt am Main, Germany. The final sample for the synchronization task included 62 participants (36 women, 23 men, 2 non-binary, 1 undisclosed gender, age range: 18 – 40 years (M = 26.28, SD = 4.16). The sample for the perception task included 57 participants (33 women, 21 men, 2 non-binary, 1 undisclosed gender, age range: 19 – 40 years (M = 26.54, SD = 4.12).
Sampling strategy	No a-priori sample size calculation was performed due to the explorative nature of the study. However, the sample size was estimated based on similar studies and stated in the preregistration.
Data collection	The stimulus presentation and response recording was performed on a Windows PC and managed with the Psychophysics Toolbox Version 3.0.12 running on MATLAB version R2021a. Questionnaire responses were collected digitally on LimeSurvey. All auditory stimuli were presented binaurally using Ethymotic Research (ER) 3c in-ear headphones with E-A-RLINK foam eartips attached to them. The whispering was recorded using a directional gooseneck condenser microphone that participants placed at around 3 cm distance from their mouth. Tapping was recorded using a microphone placed on the table. We used an audiocard (RME Fireface UC) with high precision and presented stimuli using the full duplex mode implemented in the Psychophysics Toolbox 107,108. This mode supports simultaneous sound presentation and multi-channel audio capture without any temporal jitter. We recorded the presented stimulus with a loopback microphone, which enabled us to simultaneously record the stimulus and the participant’s tapping and whispering. The researcher was not blinded about the hypotheses.
Timing	Data collection was performed from March to April 2022.

Data exclusions	As preregistered, data exclusion in the synchronization task followed the procedural recommendations for the SSS Test. Two participants were excluded because they spoke loudly instead of whispering during the synchronization task. An additional 2 participants were excluded due to inconsistency between any two trials of the same condition in the synchronization task.<br><br>In the perception task, we excluded 4 participants due to performance at or below chance level in at least one condition (stimulus x rate). Additionally, 1 participant had to be excluded due to technical problems during data acquisition.
Non-participation	No participants dropped out or declined the participation.
Randomization	Participants were not assigned in experimental groups or conditions.

Reporting for specific materials, systems and methods

We require information from authors about some types of materials, experimental systems and methods used in many studies. Here, indicate whether each material, system or method listed is relevant to your study. If you are not sure if a list item applies to your research, read the appropriate section before selecting a response.

Materials & experimental systems

n/a	Involved in the study	
☒	☐	Antibodies
☒	☐	Eukaryotic cell lines
☒	☐	Palaeontology and archaeology
☒	☐	Animals and other organisms
☒	☐	Clinical data
☒	☐	Dual use research of concern

Methods

n/a	Involved in the study	
☒	☐	ChIP-seq
☒	☐	Flow cytometry
☒	☐	MRI-based neuroimaging
